# Efficacy of adding manual therapy to hip and knee exercises in patients with patellofemoral pain syndrome: a double-blinded randomized controlled clinical trial

**DOI:** 10.1038/s41598-025-17453-9

**Published:** 2025-09-23

**Authors:** Nadia Abdo, Enas Fawzy Youssef, Mona Mohamed Ibrahim

**Affiliations:** Department of Physical Therapy for Musculoskeletal Disorders and its Surgery, Faculty of Physical Therapy, Cairo University, Cairo, Egypt

**Keywords:** Patellofemoral pain, Hip and knee stretch, Hip and knee strength, Manual therapy, Patellar mobilization, Iliotibial band release, Health care, Medical research, Signs and symptoms

## Abstract

Patellofemoral pain syndrome (PFPS) is the most common knee problem that affects the knee in adulthood. It is more common in females twice than in males. It has a multifactorial etiology. The effect of the combination of manual therapy and hip and knee exercises is unconfirmed till now. Therefore, the aim of this study is to investigate the effect of adding manual therapy to knee and hip exercises on pain intensity, self-reported and objective knee function, hip abductors, hip external rotators, and knee extensor strength in addition to calf, and hamstring flexibility in patients with PFPS. This was a double-blind study. Fifty-nine adult patients with PFPS matched with eligibility criteria were sequentially recruited from an outpatient clinic, and faculty students were enrolled in this study and randomized by a generated Excel sheet into two groups either control (30 patients) or manual therapy (29 patients) group. The control group received stretch and open and closed kinetic chain strength exercises, while the manual therapy group received the same exercises as the control plus patellar mobilization, iliotibial band release, and deep friction massage of lateral retinacula. This treatment was performed in the outpatient clinic for 12 sessions in 4 weeks. Fifty-three patients had only been analyzed at the end of treatment. The primary outcomes were knee pain intensity and self-reported knee function. The secondary outcomes were hip and knee muscle strength, calf and hamstring flexibility, and a step-down test. All outcomes were measured before and after 4 weeks of treatment immediately by a blind assessor. Comparing between and within groups were performed using the Multivariate analysis of variance (MANOVA) test. The mean age was 22.64 ± 3.71 years for control group, 21.92 ± 2.39 years for manual therapy group. Both treatment protocols showed significant reduction of pain (ascending (*p* < 0.001; CI: (14.85–39.44)), descending (*p* < 0.001; CI: (15.95–30.62)), squatting (*p* < 0.001; CI: (15.00-38.07)) and sitting (*p* < 0.001; CI: (13.49–37.87))) for control group, while (ascending (*p* < 0.001; CI: (20.37–39.07)), descending (*p* < 0.001; CI: (12.72–32.80)), squatting (*p* < 0.001; CI: (28.98–51.66)) and sitting (*p* < 0.001; CI: (16.72–42.24))) for manual therapy group, improvement in knee function (Kujala: (*p* < 0.001; CI: (-17.02—8.76))) for control group while (Kujala: (*p* < 0.001; CI: (-21.32—12.19))) for manual therapy group, hip and knee muscle strength and flexibility (*p* < 0.05). However, there were non-significant differences between groups in all dependent variables (*p* > 0.05). Manual therapy added to hip and knee exercises may have the same effect as hip and knee exercises without superiority to one of them. Manual therapy improved patients’ clinical outcomes, including pain and function disability, with a higher percentage of change than exercises only.

**Clinical trial registration**: www.clinicaltrials.gov*(NCT05665452). Date: 27/12/2022*.

## Introduction

PFPS is a common source of anterior knee pain. It is a non-traumatic diffuse anterior knee pain characterized by pain behind or around the patella. It is aggravated by activities that increase the load on the joint such as squatting, ascending, and descending stairs^1^. Its prevalence ranged between 12 and 35% of the general population^2^. It is more common in females than males by 2.23^3^. It increases the burden on patients and the healthcare system^4^. Furthermore, it increases the percentage of absenteeism from sports participation^1^.

PFPS has multifactorial causes, such as muscle weakness and biomechanical disorders that affect the lower limb’s dynamic stability, altering patellar tracking in the trochlear groove^5^. As a sequence of these multifactors, the load on the patellofemoral articular surface will increase which induces pain and dysfunction. As well as the joint position sense around the knee joint may be altered^6^. In addition to the muscular tightness of the gastrocnemius, soleus, hamstring, quadriceps^7^ and iliotibial band^8^ As well as a flexibility deficit of the lateral retinaculum^9^.

Patellofemoral joint hypomobility may occur due to muscle tightness, muscle weakness and soft tissue restriction that increase stress on the patellofemoral joint and result in PFPS. Another factor leading to joint hypomobility is a restriction in medial glide that occurs due to lateral connective tissue restriction of the patellofemoral joint that prevents the patella from returning to its normal position in the trochlear groove. This leads to malalignment of the patella, increased lateral joint angle, and stress of the patella against lateral trochlea, muscle imbalance that increases patellofemoral joint load^10^. Shen and his colleagues (2021) reported lateral tilt and lateral shift maltracking in adults with PFPS using dynamic MRI^11^. Some fibers of the iliotibial band connect with the patellar ligament. Therefore, restriction in the iliotibial band resulted in lateralization of patella^12^. Furthermore, neuroanatomical investigation of a piece of lateral retinaculum during surgery demonstrated a greater distribution of neural growth factors and nerve fibers in PFPS patients^13^. That may clarify the reason for increased pain in the lateral region in PFPS patients. As well as the need for lateral structure release and massage, and medial patellar glide.

Conservative rehabilitation of patients with PFPS includes individual or combined treatment. This treatment includes exercise, manual therapy, patellar taping or foot orthosis. Exercise therapy includes leg, knee, thigh, and trunk strength and stretch. Manual therapy consists of lumbar, patellofemoral, or tibiofemoral mobilization or manipulation, deep friction massage, ischemic pressure or muscular release^9^.

Unfortunately, PFPS is not self-limiting syndrome with a high rate of chronicity even after rehabilitation. The research on the rehabilitation of PFPS is huge. However, the chronicity rate of PFPS is high. Recent study reported that half of patients diagnosed with PFPS had unfavorable outcomes 5 to 8 years after rehabilitation^14^. Therefore, we need another algorithm to deal better with outcomes.

There was a classification for patients with PFPS; (1) overuse without other impairment, (2) coordination deficits, (3) muscle performance deficits (hip and knee muscles) and (4) mobility deficits (patellar hypomobility, and muscle tightness)^9^. In this study, the authors thought of a new combination of treatment based on classification that addressed muscle performance and mobility deficits^9^. This combination includes exercise that consists of weight-bearing and non-weight-bearing strengthening exercises that target posterolateral hip structure and anterior knee muscle. As well as stretching exercises that target hamstring, quadriceps, iliotibial band, and calf muscles. Manual therapy consists of medial patellar mobilization, lateral retinaculum deep friction massage and iliotibial band release. This combination aimed to improve pain intensity, functional disability, muscle strength and flexibility.

Literature demonstrated the effect of patellar mobilization on pain and function in cases with PFPS that received patellar mobilization compared to placebo and resulted in improving patient’s symptoms in the treatment group^13,15^. APTA guidelines did not recommend using patellar mobilization alone^9^. Studies that combined patellar mobilization with other interventions had low quality^16^ Combined with hip exercises alone^15,17^ or combined with quadriceps strength only against taping with iliotibial band stretch^18^.

Tanveer et al. (2025) demonstrated that patellar mobilization significantly improved outcomes more than tibiofemoral mobilization after 4 weeks of treatment. Yet, they did not use standard APTA guidelines of exercise therapy that combine weight-bearing and non-weight-bearing exercises^19^.

Sharma et al. (2025) showed that myofascial release of ITB had a greater effect than eccentric exercise of quadriceps after 4 weeks of treatment. Yet, they did not compare the effect of release against standard conventional physiotherapy and had a small sample size^20^.

Tells et al. (2016) combined the myofascial release of rectus femoris and tensor fascia lata to hip exercises only^21^. Other research combined deep friction message of lateral retinaculum to patellar mobilization without exercises^13^.

From previous research, the effect of combination of standard physical therapy exercises guided by APTA and manual therapy is unclear and needs to be confirmed. Therefore, there is a need for research that confirms this combination to direct therapists to select the most effective approach to rehabilitate patients with PFPS and help minimize unnecessary costs associated with ineffective treatment without compromising patients’ recovery. Therefore, the aim of study was to investigate the effect of adding manual therapy to hip and knee exercises on pain, function, strength of hip abductors, hip external rotators and quadriceps muscles, and calf and hamstring flexibility in patients with PFPS. While the study hypothesized that there would be no significant effect of adding manual therapy to hip and knee exercises on pain, function, muscle strength and muscle flexibility in patients with PFPS.

## Materials and methods

This study investigated the efficacy of adding patellar manual therapy to hip and knee exercises on pain, function, quadriceps, hip abductors and hip external rotators muscle strength, hamstring and calf muscles flexibility in patients with PFPS. This was a double-blind randomized controlled clinical trial parallel-group study conducted at the Outpatient clinic of the Faculty of Physical Therapy, Cairo University. The study was conducted between January 2023 and April 2024. The local ethics committee of faculty of Physical Therapy, Cairo University approved the study (P.T.REC/012/004020) and registered at www.clinicaltrials.gov*(NCT05665452).* All experiments were performed in accordance with relevant guidelines and regulations.

### Participants

Fifty-nine consecutive patients with patellofemoral pain syndrome were enrolled in this study, including 30 patients in the control group (21 females and 9 males) and 29 patients (23 females and 6 males) in the manual therapy group. A minimum of 26 patients for each group was needed based on sample size calculation using G*power software (version 3.1.9.7, Franz Faul, Universitat Kiel, Germany) to detect an effect size of Cohen’s d = 0.52 with 95% power (alpha = 0.05)^22^.

Patients were recruited from the outpatient clinic of Faculty of Physical Therapy of Cairo University and college students of the same faculty based on the following diagnostic criteria that performed by the primary investigator (11 year experience in physical therapy of musculoskeletal disorders): had a history of insidious onset, patellar tenderness in either medial or lateral borders, retropatellar pain, and had anterior knee pain during two or more of provocative activities that include stair ascent or descent, kneeling, prolonged sitting, or squatting and positive patellar compression test^9^. Patients with PFPS included if meeting these inclusion criteria: age ranging between 18 and 35 years^23–25^, duration of symptoms of PFPS is greater than 4 weeks^23,26,27^^,^ pain intensity is more than 3 at visual analogue scale^23,25,26^.

Patients were excluded if they had previous patellar realignment surgery or fracture^23,25^, traumatic patellar dislocation^25,28^, knee surgery^28^, knee menisci, ligaments, bursae, or synovial plica syndrome dysfunction^24,25^, any form of inflammatory arthritis that includes osteoarthritis, rheumatoid arthritis^23,25^ or gout, taking corticosteroids or nonsteroidal anti-inflammatory medication or inability to attend treatment program to the end of sessions^14^.

Recruited participants were screened against the eligibility criteria by the first author N.A (11 years of clinical experience). Participants signed informed consent after they agreed to participate. Then, basic demographic information was collected, and patients started the test. After that, patients received the treatment program.

### Treatment procedures

According to previous research^23,28^ and APTA guidelines^9^, all patients received hip and knee exercises. Stretch consisted of stretching exercises for the calf, hamstring, quadriceps, and iliotibial band. Strengthening exercises were in the form of closed (weight-bearing) and open (non-weight-bearing) kinetic chain exercises. The closed kinetic chain was in the form of minisquat, forward step up, lateral step up and terminal knee extension. At the same time, the open kinetic chain was in the form of hip abduction from side lying, hip ER from sitting and seated knee extension. In addition to hip and knee exercises, the manual therapy group received patellar mobilization, iliotibial band release, and deep friction massage of the lateral retinaculum^13,29^. All exercises were performed for 45 to 60 min per session, 3 times weekly for 4 weeks (12 sessions).

### Manual therapy

#### Medial glide patellar mobilization

**Fig. 1:**
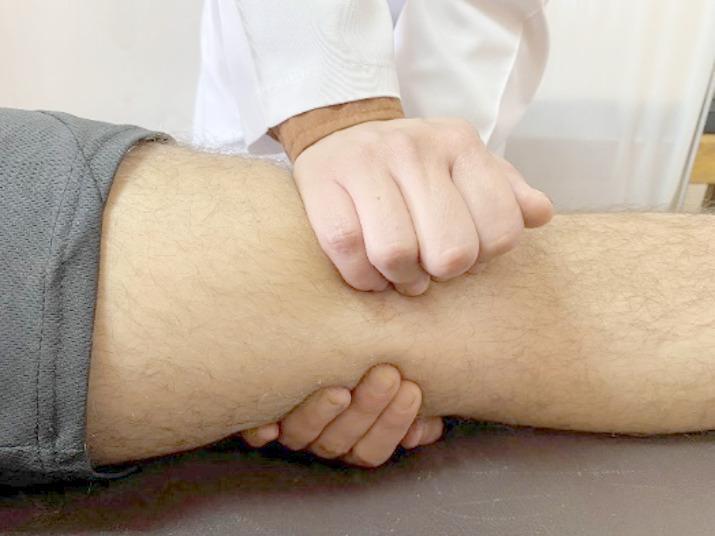
Medial glide patellar mobilization.

From a supine position with the knee extended, the therapist pushed the patella medially by the heel of the hand and sustained for one minute once as shown in Fig. 1^30^.

#### Release of the iliotibial band

**Fig. 2:**
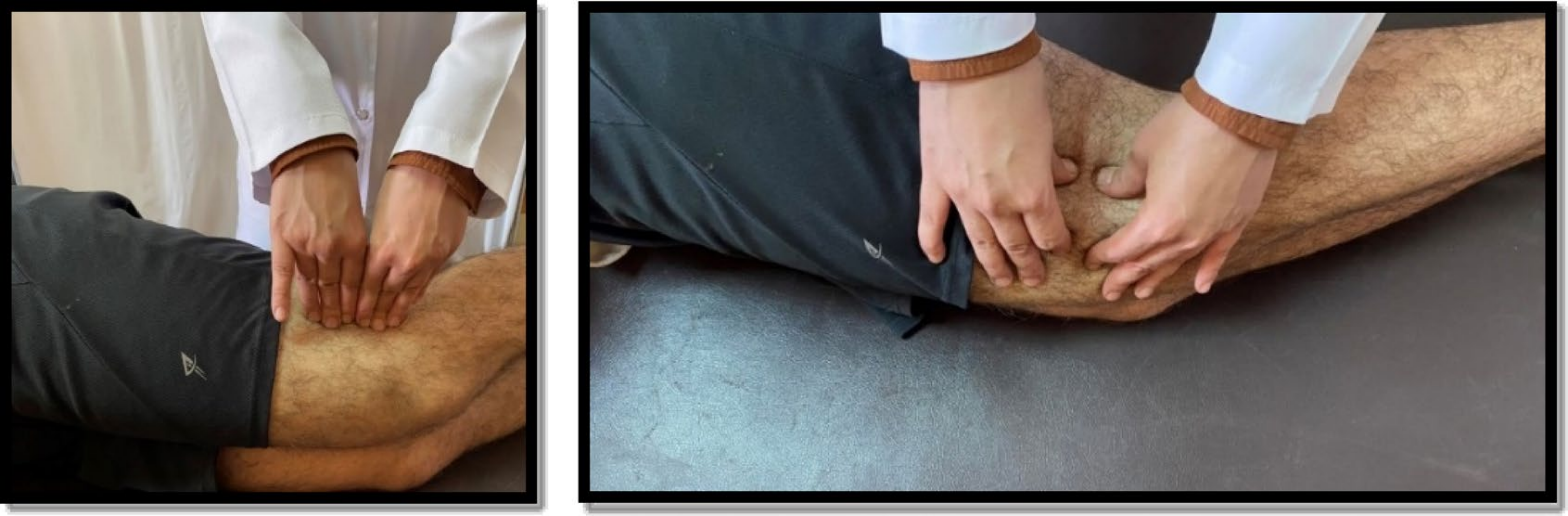
Iliotibial band release (Left), and Iliotibial band mobilization (Right).

From a supine position, the therapist put the fingers of both hands perpendicularly between ITB and vastus lateralis anterior and ITB and biceps femoris posterior and maintained pressure in each position for one minute once, as shown in Fig. 2. Then the therapist applied ITB mobilization, grasping it between the thumb and index of both hands, moving it in the anterior or posterior direction according to restrictions, and sustained for one minute one time as shown in Fig. 2^20,31^.

#### Deep friction massage of the lateral retinaculum

**Fig. 3:**
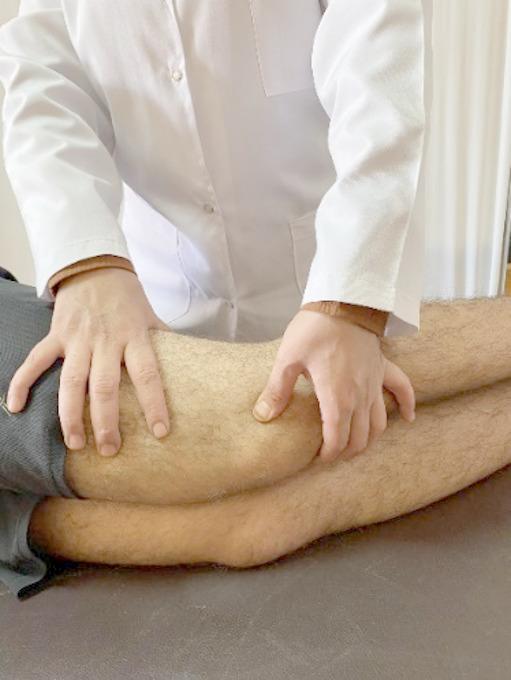
Deep friction message to the lateral retinaculum.

The patient was in a supine position, the therapist applied deep friction massage perpendicular to the retinaculum and sustained for one minute as shown in Fig. 3^13^. The progression in manual therapy depends on the occurrence of release and gaining new barriers.

**Fig. 4:**
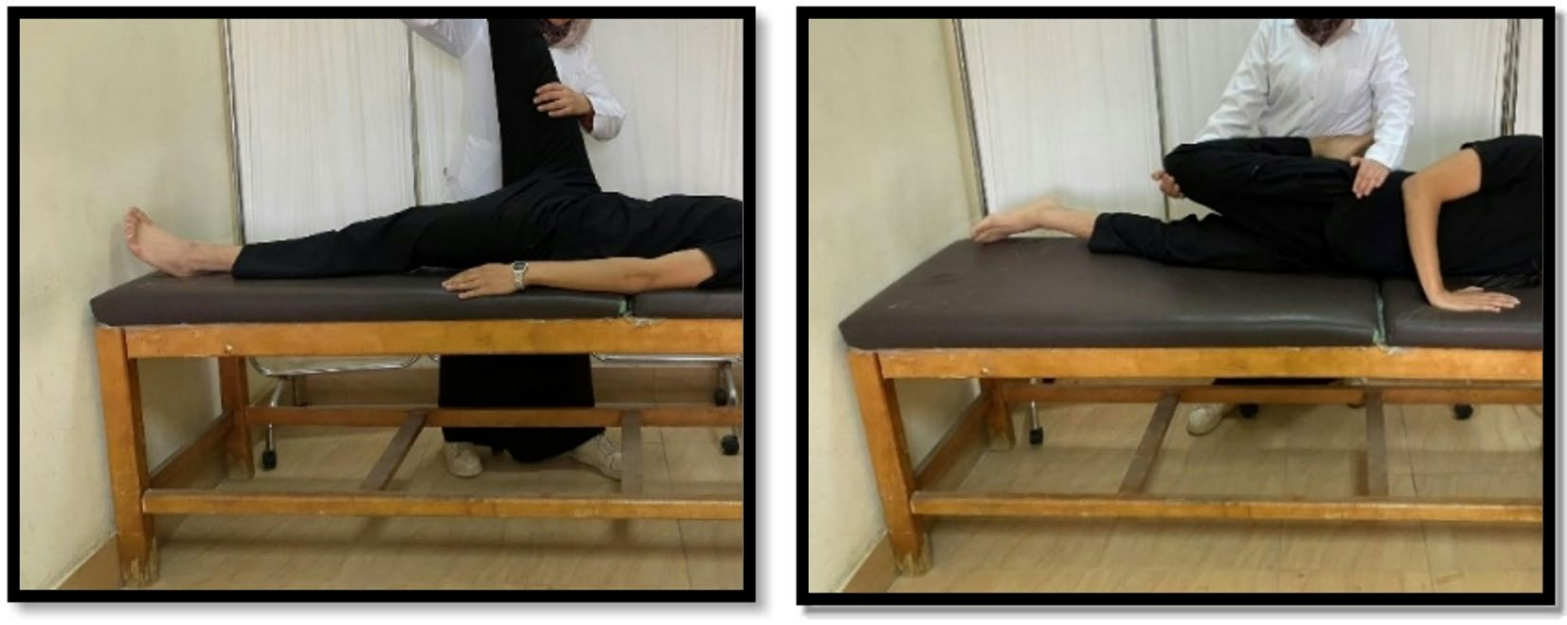
Hamstring stretch (Left), and Quadriceps stretch (Right).

**Fig. 5:**
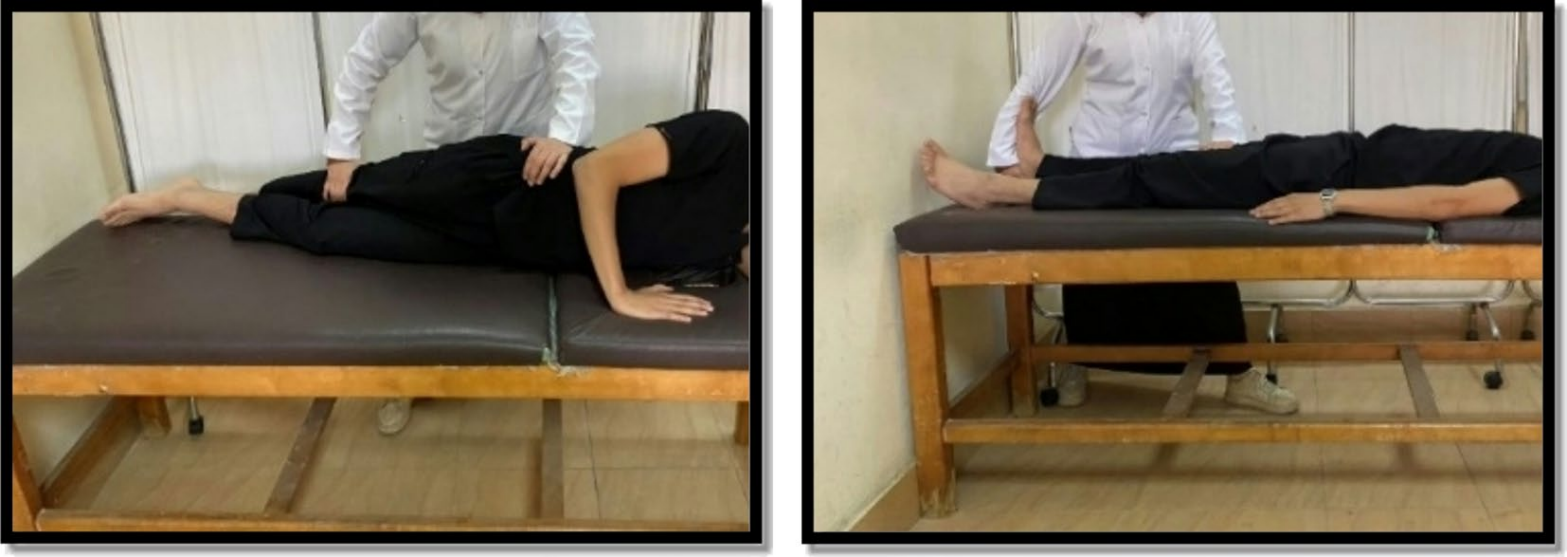
Iliotibial band stretch (Left), and Gastrocnemius stretch (Right).

Stretching exercises were conducted for the calf and hamstring from a supine position, the quadriceps, and the iliotibial band from a side-lying position. The therapist held the stretch position for 30 s for three repetitions for each muscle as shown in Figs. 4 and 5^9,23,28^.

**Fig. 6:**
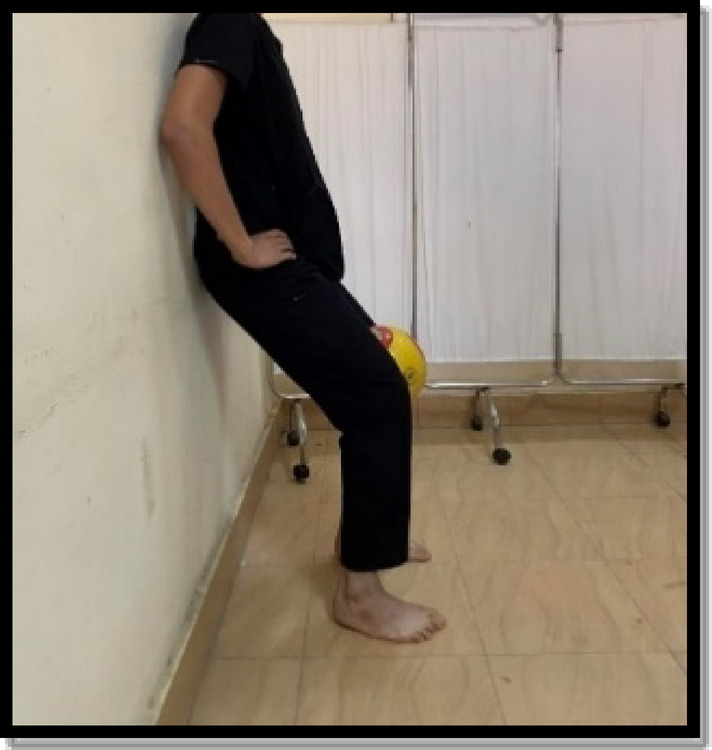
Mini wall squat exercise.

**Fig. 7:**
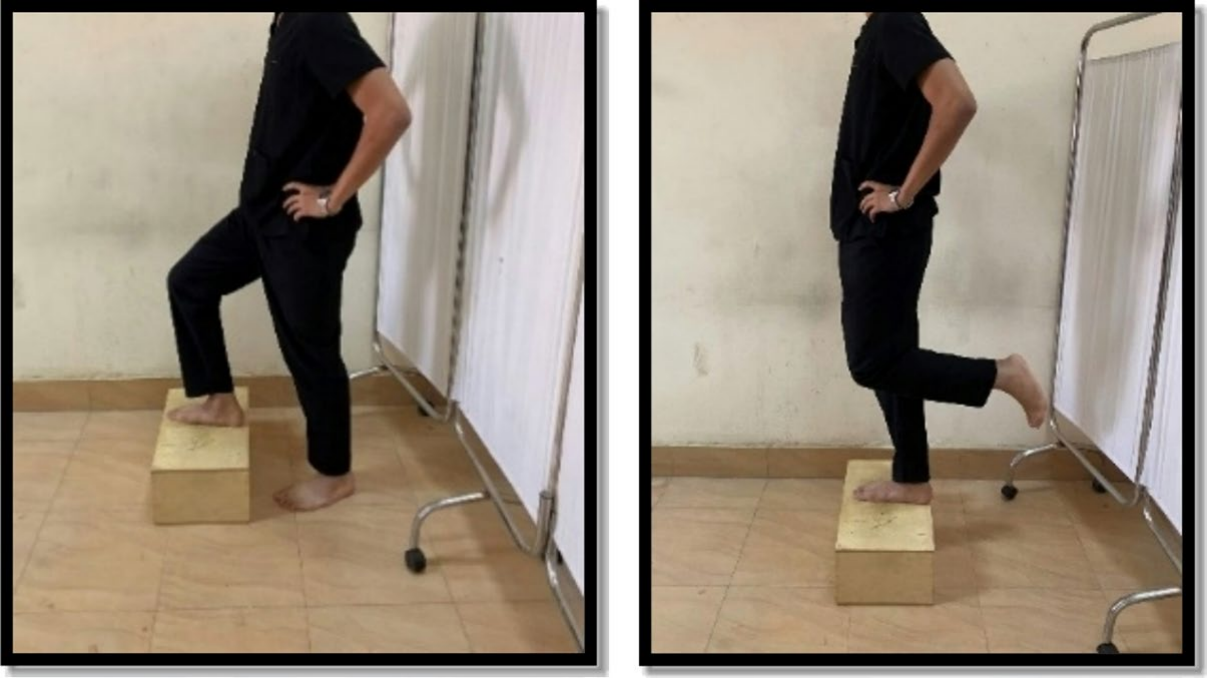
Forward step-up exercise.

**Fig. 8:**
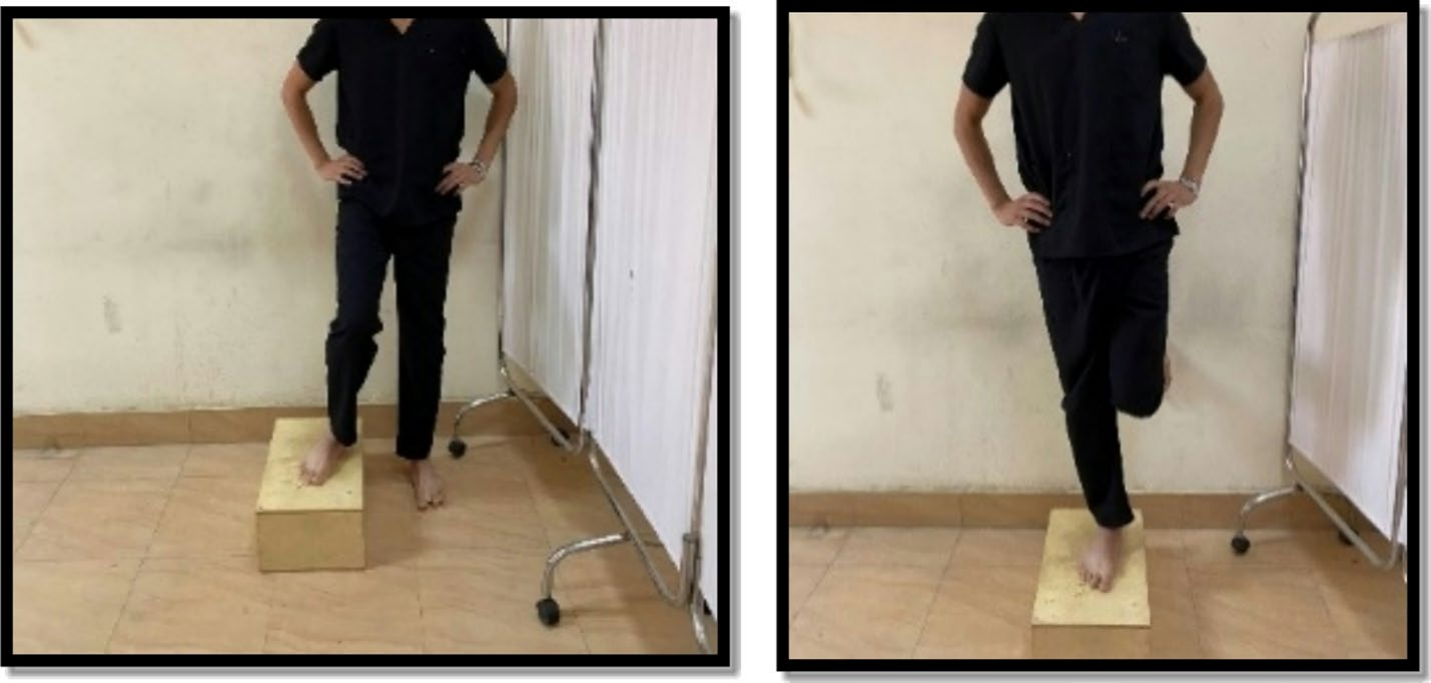
Lateral step-up exercise.

**Fig. 9:**
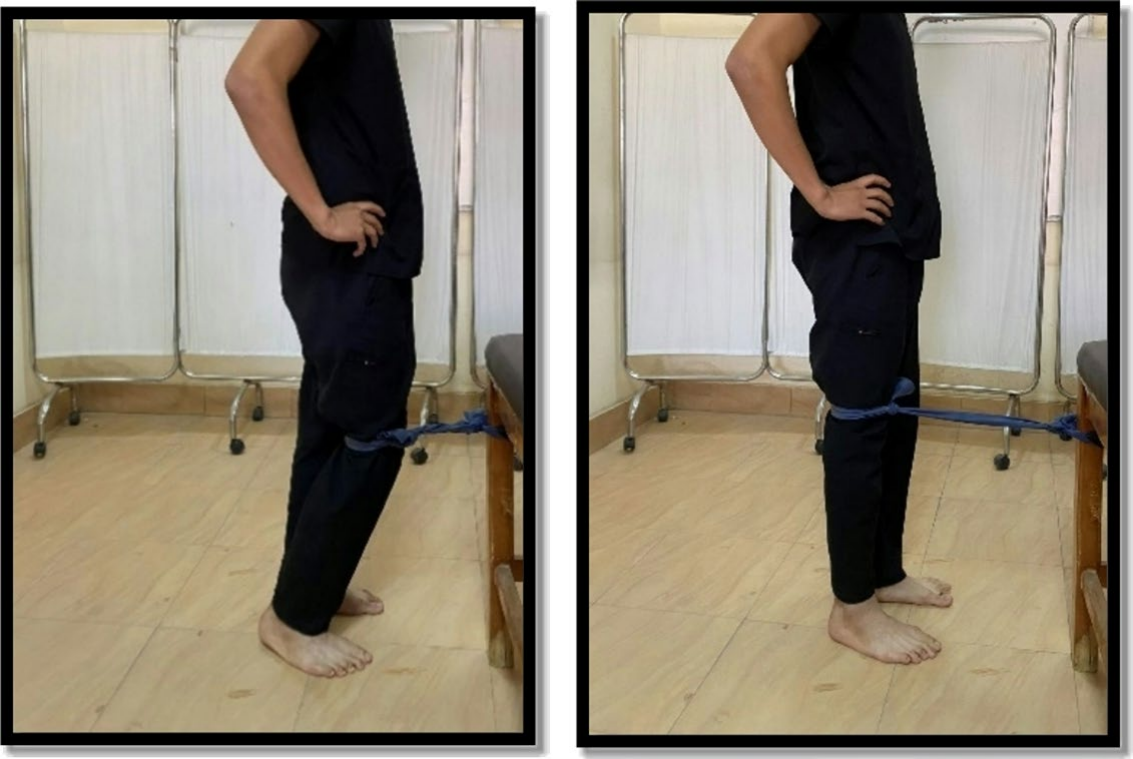
Terminal knee extension.

Strengthening exercises were in the form of closed and open kinetic chain exercises. The closed kinetic chain was a mini squat against a wall (0º-40º), forward and lateral step up, and terminal knee extension from standing. Each exercise was held for 6 s and the rest for 3 s for 10 repetitions. There was 1 min rest between exercises as shown in Figs. 6, 7, 8 and 9. Exercises were progressed weekly by the addition of 2 repetitions^9,23,28^.

**Fig. 10:**
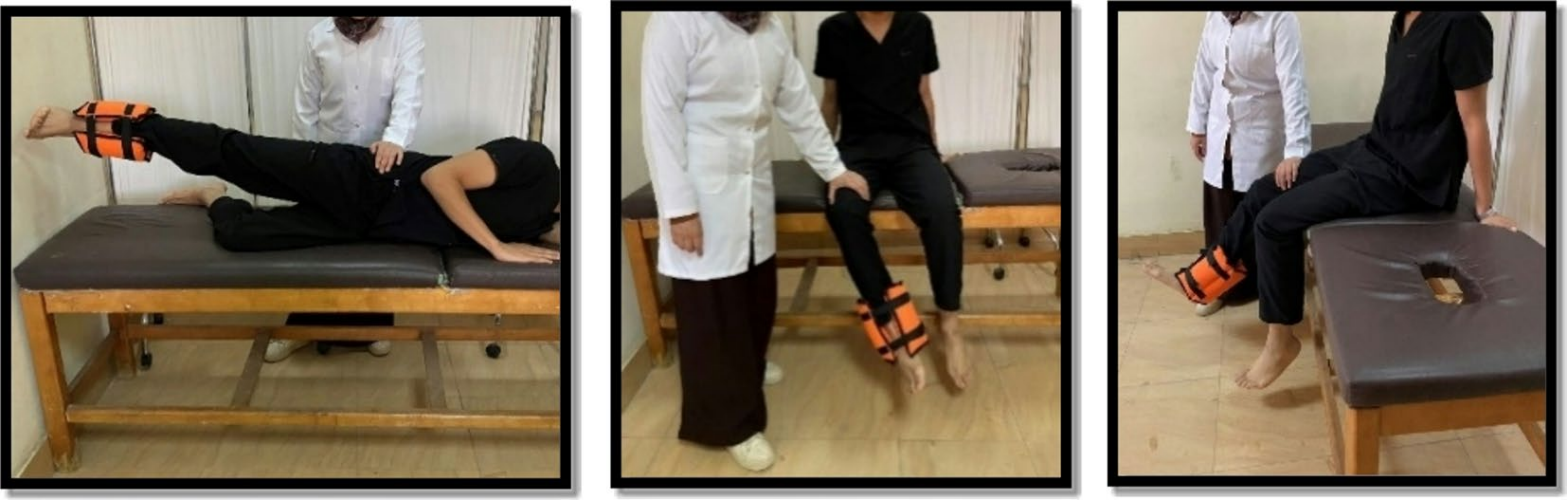
Hip abductors (Left), external rotators (center), and Knee extensors (Right) strength.

While the open kinetic chain was in the form of hip abduction from side-lying, hip ER from sitting, and seated knee extension. Each exercise was performed 10 repetitions for two sets. The patient held the position for 6 s and relaxed for 3 s between repetitions and 1 min between sets and between exercises as shown in Fig. 10^9,23,28,32,33^. The sandbag’s weight for giving resistance ranged between 0.5 and 10 KG. The exercise was progressed weekly according to 10 repetitions maximum.

### Outcome measures

All outcome measures were assessed immediately before the training program and immediately after the end of the program, and the assessment session was extended for about 45 min by blind assessor B. A (11 years of clinical experience) who was blinded to the patient’s group.

The primary outcome measures were (1) pain severity, assessed by the Visual analogue scale (VAS). It is a horizontal line between 0 and 10, where 0 represents no pain and 10 represents severe, intolerable pain^23,25^. It is a valid (*r* = 0.62–0.9)^34^ and reliable (*r* = 0.937z)^35^ scale for assessing pain severity. The patient was instructed to draw a line representing the pain experienced during stair descent, ascent, squatting, and prolonged sitting with 90-degree knee flexion^23,25^. (2) Self-reported knee function assessed using 13-item Kujala patellofemoral disorder Arabic version score which represents a self-reported functional scale for the knee joint. It includes 13 items that are representable to provocative activities in PFPS^25,36^. It has a valid S-CVI = 86.5% and a reliable (Spearman correlation between 0.7 and 0.9) Arabic version^37^. The scale ranges from 0 to 100, here 100 represents no functional limitation^23,25^.

The secondary outcome measures were (1) Hamstring flexibility assessment (90/90 test), from the supine laying, the assessor raised the hip of the involved limb to 90° flexion, then extended the knee passively and recorded the popliteal angle^38,39^. (2) Gastrocnemius flexibility assessment. The patient lay supine, and the assessor dorsiflexed the ankle joint and measured the dorsiflexion angle^40^. (3) Hip abductor muscle strength assessment, the patient was positioned on the side lying with the affected limb was the top with a pillow between both legs to maintain the hip and knee in a neutral position (0° abduction and flexion). The dynamometer was secured by two fingers proximally to the lateral malleolus^41,42^. (4) Hip external rotators muscle strength assessment. The patient was seated with 0° hip rotation and 90° knee flexion. The dynamometer was placed 2.5 cm proximally to the medial malleolus^42^. (5) Knee extensor muscle strength assessment, the patient was seated with 0° hip rotation and 60° knee flexion. The dynamometer was placed 2.5 cm proximally to the medial malleolus in front of the leg^42^. A hand-held dynamometer (Model 01165, Lafayette Instrument Company, Indiana, and USA) was used to assess muscle strength. It is a valid and reliable (*r* = 0.9) simple methodological tool to assess muscle strength. It is used in clinical and research settings^43^. For all tests of strength, the patient received encouragement to perform maximum isometric contraction. The patient performed 1 practice trial. Then, he/ she performed a testing trial for 5 s for maximum contraction. Muscle force was normalized according to this formula (muscle force Kg/body weight Kg * 100)^42,44^. **(6)** Functional performance is assessed by step-down test. It is a functional test that mimics step-down stairs used to measure the functional performance of the lower limb. It is a reliable test (ICC: 0.79 to 0.94) for assessing the functional performance in PFPS. The investigator counted the number of repetitions in 30 s^42,45^.

### Randomization

Patients were randomized to either a control group or a manual therapy group using a random computer-generated Excel sheet performed by N.A.

### Blindness

Patients were unaware of the other group’s group allocation and exercises to keep blindness. As well as the outcome measures were assessed by a blinded assessor.

### Statistical analysis

Descriptive statistics were presented as mean ± standard deviation (SD), whereas qualitative variables were expressed as a count (percentage). The Shapiro-Wilk test was used to test the normality, revealing that all dependent variables were normally distributed. In addition, homogeneity of variance was tested via Levene’s test, which revealed all data showed no violations of the assumptions of equality of variance with *p*-value ˃ 0.05. Comparing between groups and within groups were performed using the MANOVA test.

## Results

**Fig. 11:**
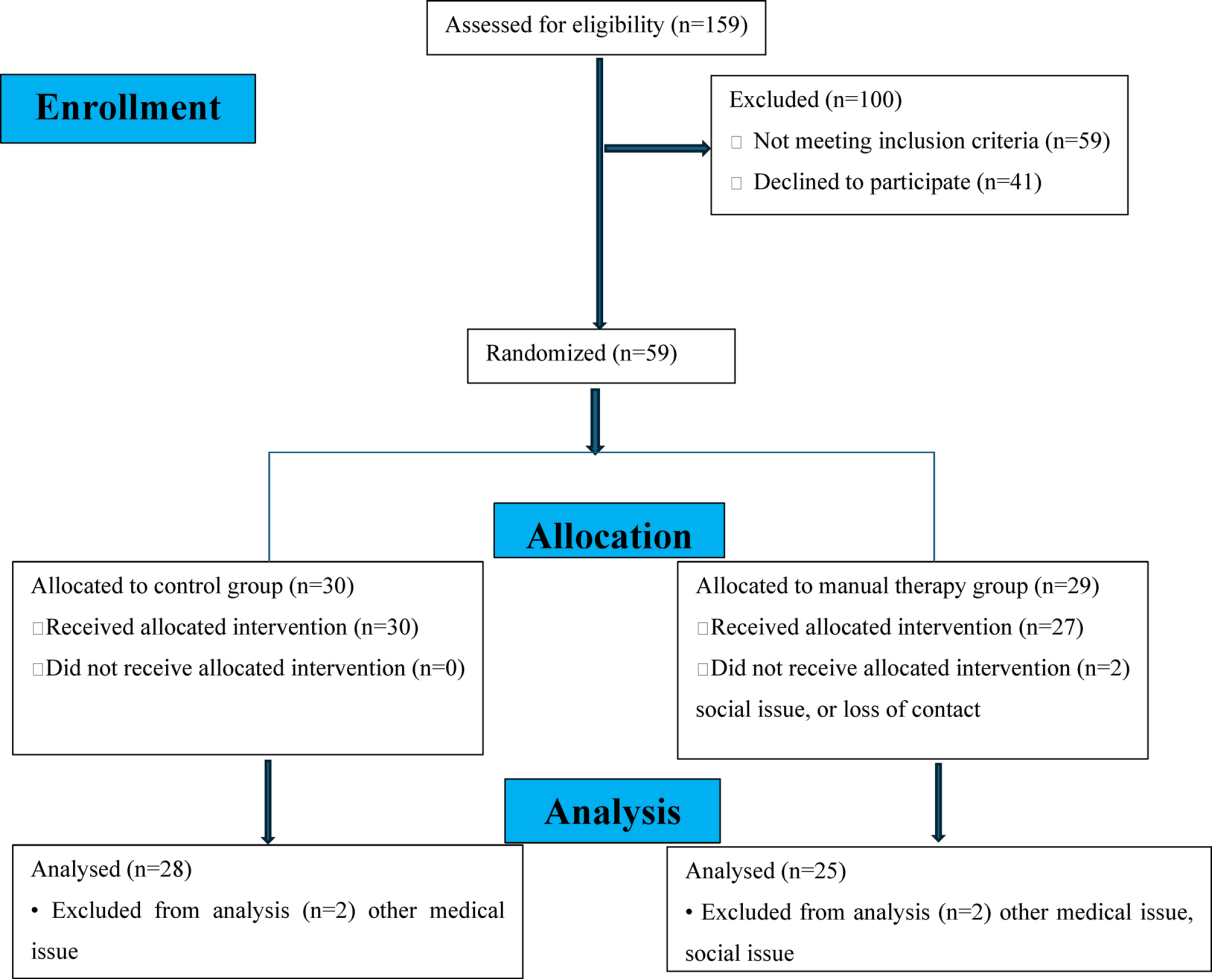
Flow chart of the study groups.

One hundred fifty-nine were screened for this study against the eligibility criteria. Fifty-nine were matched with these criteria and the remaining individuals were excluded due to inability to attend sessions, age > 35, rheumatoid arthritis, knee injury, and other causes. Fifty-seven have received the allocated intervention. At the end, 53 was analyzed as shown in the flow chart Fig. 11. Patients were 100% compliant with treatment sessions. Patients were recruited from January 2023 to April 2024. Outcome measures were assessed immediately before the beginning and after 4 weeks of treatment. Basic patients’ demographics were not statistically significant between the groups (*p* > 0.05) (Table 1).

**Table 1:** Patients’ demographics for both groups.

Variable	Mean ± SD		t-value	*P*-value	Sig.
	Control group *N* = 28	Manual therapy group *N* = 25			
Age (years)	22.64 ± 3.71	21.92 ± 2.39	0.83	0.41	NS
Weight (kg)	64.87 ± 11.51	68.06 ± 8.96	-1.11	0.27	NS
Height (cm)	167.19 ± 8.27	166.20 ± 9.51	0.41	0.69	NS
BMI (kg/m^2^)	23.18 ± 3.60	24.77 ± 3.63	-1.60	0.12	NS

The repeated measure MANOVA showed statistically significant group time interaction of both treatment groups for all dependent variables (*p* < 0.001). However, there was a non-significant difference in effect of treatment between both groups for VAS ascending (*p* = 0.74), VAS descending (*p* = 0.93), VAS squatting (*p* = 0.09), VAS Sitting (*p* = 0.66), Kujala (*p* = 0.20), Hip abductors strength (*p* = 0.84), Hip external rotators strength (*p* = 0.95), Quadriceps strength (*p* = 0.17), Hamstring flexibility (*p* = 0.82) and Calf flexibility (*p* = 0.28).

### Pain intensity

There was a significant reduction in pain level (VAS ascending, VAS descending, VAS squatting and VAS sitting) between pre and post treatment in both groups (*p* < 0.001). However, both groups had a non-significant difference (*p* > 0.05). The percentage of change was higher in the manual therapy group as shown in Table 2.

**Table 2:** Effect of timing of rehabilitation on VAS.

		Group A *N* = 28	Group B *N* = 25	Between-group comparison		
		$\overline {{\rm X}}$± SD	$\overline {{\rm X}}$± SD	MD (CI 95%)	p-value	Sig
VAS Ascending	Pre	50.39 ± 25.42	50.68 ± 23.43	-0.287 (-13.82; 13.25)	0.97	NS
	Post	23.25 ± 18.12	20.96 ± 17.16	2.29 (-7.47; 12.05)	0.74	NS
	% of change	53.86	58.64			
	MD (CI 95%)	-27.14(14.85; 39.44)	-29.72(20.37; 39.07)			
	Effect size	0.52				
	MCID	11.72				
	p-value	< 0.001*	< 0.001*			
	Sig	Sig	Sig			
VAS Descending	Pre	40.93 ± 23.36	38.28 ± 24.75	2.65(-10.62; 15.92)	0.69	NS
	Post	17.64 ± 17.79	15.52 ± 17.12	2.12(-7.54; 11.78)	0.93	NS
	% of change	56.90	59.46			
	MD (CI 95%)	-23.29(15.95; 30.62)	-22.76(12.72; 32.80)			
	Effect size	0.54				
	MCID	12.37				
	p-value	< 0.001*	< 0.001*			
	Sig	Sig	Sig			
VAS Squatting	Pre	53.43 ± 26.88	64.28 ± 28.59	-10.85(-26.16; 4.45)	0.16	NS
	post	26.89 ± 22.31	23.96 ± 18.56	2.93(-8.46; 14.34)	0.09	NS
	% of change	49.67	62.73			
	MD (CI 95%)	-26.54 (15.00; 38.07)	-40.32 (28.98; 51.66)			
	Effect size	0.59				
	MCID	13.44				
	p-value	< 0.001*	< 0.001*			
	Sig	Sig	Sig			
VAS Sitting	Pre	58.54 ± 31.55	57.16 ± 31.34	1.38(-15.99; 18.75)	0.87	NS
	post	32.86 ± 24.22	27.68 ± 25.52	5.18(-8.55; 18.89)	0.66	NS
	% of change	43.87	51.57			
	MD (CI 95%)	-25.68(13.49; 37.87)	-29.48(16.72; 42.24)			
	Effect size	0.45				
	MCID	15.67				
	p-value	< 0.001*	< 0.001*			
	Sig	Sig	Sig			

### Self-reported knee function (Kujala)

A significant improvement was found in both groups between pre- and post-rehabilitation (*p* < 0.001). But there was a non-significant difference between the groups (*p* > 0.05). The percentage of change was higher in the treatment group, as shown in Table 3.

**Table 3:** Effect of timing of rehabilitation on kujala,

		Group A *N* = 28	Group B *N* = 25	Between-group comparison		
		$\overline {{\rm X}}$± SD	$\overline {{\rm X}}$± SD	MD (CI 95%)	p-value	Sig
Kujala	Pre	70.86 ± 13.76	67.08 ± 13.56	3.78 (-3.77; 11.33)	0.32	NS
	post	83.75 ± 9.50	83.84 ± 10.63	-0.09 (-5.64; 5.46)	0.20	NS
	% of change	-18.19	-24.99			
	MD (CI 95%)	12.89 (-17.02; -8.76)	16.76 (-21.32; -12.19)			
	Effect size	0.66				
	MCID	6.78				
	p-value	< 0.001*	< 0.001*			
	Sig	Sig	Sig			

### Muscle strength

Both groups had a significant difference in hip abductors, hip external rotators, and knee extensor muscle strength between pre- and post-rehabilitation (*p* < 0.001). There was a non-significant difference between both groups in all muscle strength (*p* > 0.05) with a higher percentage of change in the manual therapy group, except in knee extensor strength, which was higher in the control group, as shown in Table 4.

**Table 4:** Effect of timing of rehabilitation on hip abductor strength, hip external rotator strength and knee extensor strength.

		Group A *N* = 28	Group B *N* = 25	Between-group comparison		
		$\overline {{\rm X}}$± SD	$\overline {{\rm X}}$± SD	MD (CI 95%)	p-value	Sig
Hip Abductors strength	Pre	14.83 ± 5.66	13.15 ± 4.46	1.69 (-1.15; 4.52)	0.24	NS
	post	17.51 ± 4.88	16.08 ± 5.39	1.44 (-1.39; 4.27)	0.84	NS
	% of change	-18.07	-22.28			
	MD (CI 95%)	2.68 (-4.50; -0.86)	2.93 (-4.75; -1.11)			
	Effect size	0.28				
	MCID	2.23				
	p-value	< 0.001*	< 0.001*			
	Sig	Sig	Sig			
Hip External rotator strength	Pre	13.34 ± 5.02	12.01 ± 4.72	1.33 (-1.36; 4.03)	0.33	NS
	post	15.28 ± 4.93	13.88 ± 6.38	1.39 (-1.72; 4.52)	0.95	NS
	% of change	-14.54	-15.57			
	MD (CI 95%)	1.94 (-3.44; − 0.45)	1.88 (-3.49; -0.25)			
	Effect size	0.20				
	MCID	2.36				
	p-value	< 0.001*	< 0.001*			
	Sig	Sig	Sig			
Knee extensor strength	Pre	23.81 ± 9.76	25.74 ± 11.22	-1.93 (-7.72; 3.85)	0.51	NS
	post	31.41 ± 12.07	30.28 ± 12.01	1.13 (-5.52; 7.78)	0.17	NS
	% of change	-31.92	-17.64			
	MD (CI 95%)	7.6 (-11.13; -4.08)	4.54 (-7.32; -1.76)			
	Effect size	0.37				
	MCID	5.61				
	p-value	< 0.001*	< 0.001*			
	Sig	Sig	Sig			

### Muscle flexibility

A significant difference was found in hamstring and calf flexibility between pre- and post-rehabilitation in both groups (*p* < 0.001). A non-significant difference was found between both groups in both muscle’s flexibility (*p* > 0.05). The percentage of change was higher in the control group in hamstring flexibility, while higher in the manual therapy group in calf flexibility, as shown in Table 5.

**Table 5:** Effect of timing of rehabilitation on hamstring flexibility and calf flexibility.

		Group A *N* = 28	Group B *N* = 25	Between-group comparison		
		$\overline {{\rm X}}$± SD	$\overline {{\rm X}}$± SD	MD (CI 95%)	p-value	Sig
Hamstring flexibility	Pre	44.86 ± 13.67	51.05 ± 11.76	-6.19 (-13.26; 0.89)	0.09	NS
	post	55.61 ± 10.27	60.95 ± 12.59	-5.34 (-11.57; 0.88)	0.82	NS
	% of change	-23.96	-19.39			
	MD (CI 95%)	10.75 (-15.61; -5.87)	9.89 (-15.66; -4.13)			
	Effect size	0.39				
	MCID	5.88				
	p-value	< 0.001*	< 0.001*			
	Sig	Sig	Sig			
Calf flexibility	Pre	37.31 ± 9.91	33.82 ± 8.22	3.49 (-1.57; 8.54)	0.17	NS
	post	40.48 ± 8.51	39.53 ± 6.73	0.95 (-3.32; 5.22)	0.28	NS
	% of change	-8.49	-16.88			
	MD (CI 95%)	3.17 (-6.55; 0.22)	5.71 (-9.09; -2.31)			
	Effect size	0.22				
	MCID	4.11				
	p-value	< 0.001*	< 0.001*			
	Sig	Sig	Sig			

### Functional performance test

There was a significant improvement in the step-down test in both groups, with a higher percentage of change in the control group as shown in Table 6.

**Table 6:** Effect of timing of rehabilitation on step-down test.

		Group A *N* = 28	Group B *N* = 25	Between-group comparison		
		$\overline {{\rm X}}$± IQR	$\overline {{\rm X}}$± IQR	χ2 value	p-value	Sig
Step-down test	Pre	12 ± 5	13 ± 4	28.23	0.013	Sig
	post	15 ± 5	16 ± 4	27.09	0.019	Sig
	% of change	-25	-23.08			
	MD	3	3			
	Effect size	0.35				
	MCID	1.47				
	Sig	Sig	Sig			

## Discussion

This study investigates the effect of adding manual therapy to hip and knee exercises on the intensity of pain measured by VAS, self-reported function measured by Kujala, hip abductors, hip external rotators, and knee extensor strength measured by handheld dynamometer, calf and hamstring flexibility measured by universal goniometer and objective function using step-down test. As well as comparing the results of the manual therapy group to those of the control group.

The results showed a statistically significant reduction in pain, improvement in objective and self-reported function, hip abductors, hip external rotators, and knee extensor strength as well as improvement in hamstring and calf flexibility in both groups, with a higher percentage of change in the manual therapy group. However, the groups had no significant differences in all dependent variables.

These results are consistent with those of Khan et al., (2024), who compared the effect of patellar mobilization and taping in addition to conventional therapy on pain measured by VAS in PFPS patients. They found a reduction in pain in both groups without significant differences between them^18^. This was also consistent with Tanveer et al. (2025) who compared the effect of patellar mobilization plus conventional physical therapy exercises with tibiofemoral mobilization plus conventional physical therapy exercises. They found an improvement in pain, function, and knee range of motion after 4 weeks of treatment. Yet, there was a greater significant improvement in patellar mobilization group^19^.

The result was also consistent with Telles et al., (2016), who demonstrated the effect of adding rectus femoris and tensor fascia lata myofascial release to hip exercises on pain measured by the numeric pain rating scale and knee function measured by the Lower Extremity Function Scale in patients with PFPS. There was a reduction in pain in both groups^21^. However, there was an improvement in function only in the myofascial group. This was also agreed with Sharma et al. (2025), who compared the effect of myofascial release of ITB with eccentric quadriceps exercise in management of patients with patellofemoral pain syndrome. They found a reduction in pain and improvement in function disability in both groups after 4 weeks of treatment. However, there were significant differences in favor of the myofascial release group^20^.

Khuman et al., (2012) compared the effect of adding patellar taping versus patellar mobilization in addition to conventional therapy in both groups on pain measured by VAS and function measured by functional outcome of knee (KOSADLS). It was consistent with the results of this study in the reduction in pain and improvement in knee function in both groups. However, there was a higher improvement in the taping group^46^.

On the other hand, van den Dolder and Roberts, (2006) found that significant increase in the number of steps and a small improvement in pain (measured by the patellofemoral pain severity questionnaire) that was insufficient to be significant after six sessions of manual therapy comparing these results with control group that did not receive any treatment^13^.

This was in contrast with the results of Patle and Bhave (2015), who investigated the effect of adding manual therapy to a supervised protocol on pain measured by VAS and activity measured by step up and step down after 2 weeks of treatment. They found improvement in outcomes in both groups, with statistically significant improvement in the experimental group compared to the control group^16^. This also contradicts Crossley et al., (2002) who demonstrated significant improvement in function measured by AKPS, an increase in the number of steps, and in usual, and worst pain measured by VAS in the treatment group compared to the placebo group, after six sessions of physiotherapy once weekly. The treatment group received hamstring and iliopsoas stretch, deep friction massage of lateral structures, medial tilt and glide of the patella, patellar taping, vastus medialis training, and isometric hip abductors^29^. Adding the patellar taping to mobilization in the same group did not clear the effect of treatment because of any technique.

Improvement in study outcomes may result from neurophysiological and mechanical effects of patellar mobilization to reduce pain through stimulation of mechanoreceptors that inhibit the spinal cord from receiving nociceptive stimulation and improve synovial fluid circulation and wash nutrients that cause pain. In addition, it stretches the shortened soft tissue and capsules around the joint to improve joint hypomobility^30^. Also, Soft tissue release and massage can realign immature scar soft tissue fibers oriented in all directions to be oriented in one direction, resulting in increased joint mobility^13^. As well as the aim of using manual therapy in the treatment is to reduce facial and muscle tension and to improve joint mobility^9^.

Eckenrode et al., (2018) reported that manual therapy applied to the knee joint had a short-term effect on pain and function when compared to control or sham therapy^47^. However, Collins et al., (2018) did not recommend the use of manual therapy alone in the conservative therapy of PFPS^48^.

Barton et al., (2015) reported that PFPS is multifactorial therefore, patients must have individually tailored multimodal treatment. Multimodal approaches include quadriceps, hamstrings, calf stretch, patellofemoral joint mobilization, and gluteal and quadriceps strength which had moderate to large effects in the short term and small effects in the long term^49^.

The systematic review of Espí-López et al., (2017) reported that manual therapy in the form of joint mobilization and soft tissue mobilization improves function and reduces pain especially if conducted with hip and knee exercises^50^. This is consistent with this study’s results and the 2018 consensus statement results^48^. As well as consistent with the systematic review of Jayaseelan et al., (2018), who reported that manual therapy improved patient symptoms compared to the control group, but the higher effect came when used in comprehensive treatment^51^.

There were no considerable differences in the results of the current study between the control and manual therapy groups. This may be because the control group was active and received treatment according to APTA guidelines, with a short period of follow-up, which could induce changes in the results of dependent variables that are not clear. Previous studies did not cover other assessment variables because we performed a comprehensive assessment according to APTA guidelines^9^.

### Limitations

This study did not measure patellar mobility before the beginning and after the end of treatment.

Furthermore, this study did not contain people with high levels of physical activity who were affected by PFPS. Therefore, it will be generalized only to sedentary and regularly physically active patients.

Therefore, it is recommended to incorporate this treatment program for a longer time with an appropriate follow-up period and add patellar movement measurements before and after the treatment.

## Conclusion

Manual therapy added to hip and knee exercises reduces pain and improves function and hip and knee muscle strength and flexibility. This is the same effect of hip and knee exercises, with a high percentage of change in favor of the manual therapy group.

## Data Availability

No datasets were generated or analysed during the current study.

## Abbreviations

PFPS: patellofemoral pain syndrome
VMO: The vastus medialis obliques
ITB: Iliotibial band release
ER: External rotation
APTA: American physical therapy association
AKPS: Anterior knee pain scale
VAS: Visual analogue scale
KOSADLS: Knee Outcome Survey Activities of Daily Living Scale
MANOVA: Multivariate analysis of variance
